# Characteristics of Anaplastic Oligodendrogliomas Short-Term Survivors: A POLA Network Study

**DOI:** 10.1093/oncolo/oyac023

**Published:** 2022-03-05

**Authors:** Louis Garnier, Chrystelle Vidal, Olivier Chinot, Elisabeth Cohen-Jonathan Moyal, Apolline Djelad, Charlotte Bronnimann, Lien Bekaert, Luc Taillandier, Jean-Sébastien Frenel, Olivier Langlois, Philippe Colin, Philippe Menei, Frédéric Dhermain, Catherine Carpentier, Aurélie Gerazime, Elsa Curtit, Dominique Figarella-Branger, Caroline Dehais, François Ducray

**Affiliations:** 1 Department of Neuro-Oncology, East Group Hospital, Hospices Civils de Lyon, Lyon, France; 2 Department of Clinical Investigation Centre (CIC-1431), Inserm, University Hospital, Besançon, France; 3 Department of Neuro-Oncology, AP-HM, University Hospital Timone, Marseille, France; 4 Department of Radiotherapy, Claudius Regaud Institut, Cancer University Institut of Toulouse, Oncopole 1, Paul Sabatier University, Toulouse III, Toulouse, France; 5 Department of Neurosurgery, University Hospital of Lille, Lille, France; 6 Department of Medical Oncology, University Hospital of Bordeaux, Bordeaux, France; 7 Department of Neurosurgery, University Hospital of Caen, Caen, France; 8 Department of Neuro-Oncology, University Hospital of Nancy, Nancy, France; 9 Department of Medical Oncology, West Cancerology Institut René Gauducheau, Saint Herblain, France; 10 Department of Neurosurgery, University Hospital of Rouen, Rouen, France; 11 Department of Radiotherapy, Courlancy Institut of Cancer, Reims, France; 12 Department of Neurosurgery and Cancerology research center, University Hospital of Angers, Angers, France; 13 Department of Radiotherapy, Gustave Roussy University Hospital, Villejuif, France; 14 Department of Neurology 2-Mazarin, APHP, University Hospital Pitié Salpêtrière-Charles Foix, Paris, France; 15 Department of Medical Oncology, University Hospital of Besançon, Besançon, France; 16 Aix-Marseille Univ, APHM, CNRS, INP, Inst Neurophysiopathol, CHU Timone, Service d’Anatomie Pathologique et de Neuropathologie, Marseille, France; 17 Cancer Initiation and Tumoral Cell Identity Department, Cancer Research Centre of Lyon (CRCL) INSERM 1052, CNRS 5286, University Claude Bernard Lyon I, Lyon, France

**Keywords:** anaplastic oligodendroglioma, age, seizure, Karnofsky Performance Status, proliferation, radiotherapy, chemotherapy, surgery

## Abstract

**Background:**

Anaplastic oligodendrogliomas *IDH*-mutant and 1p/19q codeleted (AO) occasionally have a poor outcome. Herein we aimed at analyzing their characteristics.

**Methods:**

We retrospectively analyzed the characteristics of 44 AO patients with a cancer-specific survival <5 years (short-term survivors, STS) and compared them with those of 146 AO patients with a survival ≥5 years (classical survivors, CS) included in the POLA network.

**Results:**

Compared to CS, STS were older (*P* = .0001), less frequently presented with isolated seizures (*P* < .0001), more frequently presented with cognitive dysfunction (*P* < .0001), had larger tumors (*P* = .= .003), a higher proliferative index (*P* = .= .0003), and a higher number of chromosomal arm abnormalities (*P* = .= .02). Regarding treatment, STS less frequently underwent a surgical resection than CS (*P* = .= .0001) and were more frequently treated with chemotherapy alone (*P* = .= .009) or with radiotherapy plus temozolomide (*P* = .= .05). Characteristics independently associated with STS in multivariate analysis were cognitive dysfunction, a number of mitosis > 8, and the absence of tumor resection. Based on cognitive dysfunction, type of surgery, and number of mitosis, patients could be classified into groups of standard (18%) and high (62%) risk of <5 year survival.

**Conclusion:**

The present study suggests that although STS poor outcome appears to largely result from a more advanced disease at diagnosis, surgical resection may be particularly important in this population.

## Teaser

Anaplastic oligodendrogliomas (AO) are rare brain tumors, accounting for approximately 5% of adult gliomas and 0.5% of all primary tumors affecting the central nervous system. This article reports results of a study conducted within the frame of the French POLA network on the characteristics of survivors of AO, comparing populations with a disease-specific survival of less than 5 years with populations with a survival 5 years or more.

**Implications for Practice**

Although anaplastic oligodendrogliomas are typically associated with a prolonged survival, approximately 20% of patients have a poor outcome and a survival inferior to 5 years. The present study demonstrates that these patients present aggressive baseline characteristics, highlights features that could enable their identification, and suggests an important role of surgical resection in these patients.

## Introduction

Anaplastic oligodendrogliomas (AO) are rare brain tumors, accounting for approximately 5% of adult gliomas and 0.5% of all primary tumors affecting the central nervous system.^1^ They are defined by the 2016 World Health Organization (WHO) classification^2^ as *IDH*-mutant 1p/19q-codeleted diffuse gliomas with increased mitotic activity, microvascular proliferation, and/or necrosis.^3^ Among high-grade gliomas, AO have a better prognosis than high-grade *IDH*-mutant astrocytomas and a much better prognosis than *IDH*-wild-type glioblastomas.^3^ Standard treatment consists of maximal safe surgical resection followed by radiotherapy plus PCV chemotherapy (CT) regimen (procarbazine, CCNU [lomustine], and vincristine).^4,5^ Anaplastic oligodendrogliomas are typically associated with a prolonged survival with a median survival estimated to approximately 15 years.^4,5^ However, approximately 20% of patients have a poor outcome and survive less than 5 years.^4-6^ Poor prognostic factors have been identified in AO,^7-9^ yet the characteristics of AO short-term survivors (STS) remain to be described.^10^ The aim of the present study was to analyze the characteristics of STS, defined as patients with a disease-specific survival <5 years and to compare with those of AO patients with a survival ≥5 years (classical survivors, CS). It was conducted within the frame of the French POLA network dedicated to anaplastic oligodendroglial tumors.

## Material and Methods

### POLA Network and Patients

In 2008, the French *Institut National du Cancer* supported the creation of a national network named “*Prise en charge des OLigodendrogliomes Anaplasiques*” (POLA). This network prospectively collects samples, characteristics, and outcomes of patients diagnosed with high-grade oligodendroglial tumor in French academic centers. Among the 2189 patients included in the POLA network, patients with centrally reviewed confirmation of newly diagnosed AO were prospectively included in the present study. Formalin-fixed paraffin-embedded (FFPE) tumor tissue was available for pathological and immunohistochemical investigations for all cases. Patients provided written informed consent for clinical data collection and genetic analysis according to national and POLA network policies. We retrospectively analyzed data from all patients registered in the POLA network from 2008 to 2019. The following clinical data were collected: age at diagnosis, preoperative symptoms and Karnofsky Performance Status (KPS), surgical and postoperative treatments, tumor location and characteristics, survival status, and survival time. Extent of resection (EOR) was recorded as biopsy or resection. The initial postoperative treatment strategy was classified as radiotherapy (RT) alone, CT alone, RT + CT (sequential and/or concurrent), simple follow-up, and/or no treatment. CT regimen was defined as PCV, TMZ (temozolomide), or CCNU (lomustine). Tumor volumes could be measured for a subset of 64 patients and were calculated according to the 3 largest diameter technique using T2 or FLAIR-weighted magnetic resonance imaging.^11^

### Pathological Review and Immunohistochemistry

All cases of supposed AO were centrally reviewed and included in the prospective POLA network if they met the pathological inclusion criteria of AO according to the WHO classification of brain tumors.^2^ The presence of mitoses (with mitotic index referring to the number of mitotic figures per 10 High Power Fields), marked atypia, areas of high cellularity, microvascular proliferation, and necrosis were assessed. In addition, automated immunohistochemistry was performed on 4-µm-thick FFPE sections with avidin-biotin-peroxydase complex on Benchmark XT (Ventana Medical System Inc., Tucson, AZ, USA) using the Ventana Kit including DAB reagent to search for expression of IDH1 R132H (clone H09; 1:75; Diavona), p53 (clone DO.7; 1:200; Dako), ATRX (polyclonal; Sigma), Ki67/MIB1 (clone Mib1;:100; Dako), EGFR (clone EGFR.25; 1:100; BNovocasta), and inactivating mutations in the transcriptional repression factor Capicua (*CIC*). EGFR positive expression was assessed using the Hirsch score as previously described.^12^ P53-positive expression was considered with a cutoff at 10%.

### DNA Extraction, Single Nucleotide Polymorphism Array, and Comparative Genomic Hybridization Array Procedures

Following the manufacturer’s recommendations, tumor DNA was extracted from frozen tissue, or FFPE samples using the iPrep ChargeSwith Forensic Kit. Qualification and quantification of tumor DNA were fulfilled using a NanoVue spectrophotometer and gel electrophoresis, respectively. When necessary, the genomic profile was assessed using single nucleotide polymorphism (SNP) or CGH arrays, as described previously.^13^*TERT* mutation were also assessed as previously described.^14^

### Statistical Analysis

SNP and CGH array analysis were performed as previously described.^15^ For arrays, genomic imbalances were classified as loss, gain, homozygous deletion, or amplification. For correlation between chromosomal arm imbalances and histological variables, the Fisher’ exact test (for factors) or the Student’s *t* test (for quantitative variables, when they were scored as positive or negative) were used. Continuous variables were compared using Mann-Whitney *U* test. Overall survival (OS) was defined as the time from surgery to tumor-progression-related death (patients who died from other causes were excluded from the retrospective analyses). Progression-free survival (PFS) was defined as the time from surgery to first progression or last follow-up in case of unprogressive tumor. In order to identify clinical, radiological, pathological, and/or genomic factors related to OS, survival curves were obtained according to the Kaplan-Meier method and compared using the log-rank test for univariate comparisons. Cox proportional hazards models were used for multivariate analyses and for estimating hazard ratios in survival regression models. Because of the large number of potiential explanatory variables, multivariate analysis only included all the variables with a *P*-value of <.02 in univariate analyses. All variables obtained were searched for prognostic significance. The final model was fit using a backward method of selection. All statistical tests were 2-sided, and the final threshold for statistical significance was *P*-value = .05. Analysis was performed by the Clinical Investigation Center (Inserm CIC 1431) of Besançon and was conducted using SAS for windows version 9.4.

## Results

### Patient Selection

At the time of analysis, among the 519 AO patients included in the POLA network, 318 patients were alive and their follow-up was <5 years, 146 patients had a survival ≥ 5 years (and constituted the CS group) and 55 patients had a survival < 5 years. Among the latter patients, 44 patients (80%) died from tumor progression and 11 patients (20%) died from another cause (suicide *n* = 2, other cancer *n* = 2, post-operative cerebral hemorrhage *n* = 1, stroke *n* = 1, congestive heart failure *n* = 1, pulmonary embolism *n* = 1, aortic aneurysm rupture *n* = 1, sepsis *n* = 1, car crash *n* = 1), while their last evaluation indicated stable disease. We considered as AO STS the 44 patients with a disease-specific survival < 5 years. Patients who died from another cause than tumor progression were excluded from the analysis. The CONSORT flow diagram of patient selection for the study cohort is available in Supplementary Fig. 1. The median survival of STS patients was 2 years, the median survival of CS patients was not reached after a median follow-up of 7 years. The median PFS was 0.68 years for STS patients and 5 years in CS patients.

### Clinical and Imaging Characteristics

The clinical and imaging characteristics of STS and CS are summarized in Table 1. Compared to CS, STS were older at diagnosis (median 57.4 vs 48.1 years; *P* = .= .0001), had a poorer pre-operative KPS (KPS < 80) (43.2% vs 67.1%; *P* = .= .005), more frequently presented with focal deficits (27.3% vs 11%; *P* = .= .02), intracranial hypertension (52.3% vs 29.5%; *P* = .= .0039), and cognitive dysfunction (59.1% vs 17.8%; *P* < .0001). They less frequently presented with seizures (36.4% vs 66.4% *P* = .= .0004), especially with isolated seizures (15.2% vs 58.6%, *P* < .0001).

**Table 1.** Comparison of STS and CS patients: clinical and imaging characteristics.

	All patients, *n* (%)	STS,* n* (%)	CS,* n* (%)	*P*-values^a^
*N*	190	44	146	
Age at diagnosis (median)	50.2	57.4	48.1	.0001
<40 year	41 (21.6%)	4 (9.1%)	37 (25.2%)	*ref*
[4**0-60] year**	106 (55.8%)	23 (52.3%)	83 (56.8%)	NS
>60 y**ear**	43 (22.6%)	17 (38.6%)	26 (7.8%)	.0018
≤60 y**ear** vs > 60 y**ear**				.0047
Sex				
Female	77 (40.5%)	15 (34%)	62 (42%)	*ref*
Male	113 (59.5%)	29 (66%)	84 (58%)	NS
Sex ratio M:F	1.5	1.9	1.3	
Preoperative KPS				
<80%	73 (38.4%)	25 (56.8%)	48 (32.9%)	*ref*
≥80%	117 (61.6%)	19 (43.2%)	98 (67.1%)	.005
Preoperative symptoms				
Seizures	113 (59.5%)	16 (36.4%)	97 (66.4%)	.0004
Isolated seizures^b^	73 (38.4%)	5 (15.2%)	68 (58.6%)	<.0001
Intracranial hypertension	66 (34.7%)	23 (52.3%)	43 (29.5%)	.0039
Speech disorder	11 (5.8%)	4 (9.1%)	7 (4.8%)	NS
Cognitive dysfunction	52 (27.4%)	26 (59.1%)	26 (17.8%)	<.0001
Focal deficits	28 (14.7%)	12 (27.3%)	16 (11%)	.02
Mnesic dysfunction	27 (14.2%)	10 (22.7%)	17 (11.6%)	NS
Tumor location				
Frontal	150 (78.9%)	36 (81.82%)	114 (78.1%)	NS
Temporal	45 (23.7%)	14 (31.8%)	31 (21.2%)	NS
Parietal	43 (22.6%)	17 (38.6%)	26 (17.8%)	.005
Occipital	19 (10%)	8 (18.2%)	11 (7.5%)	.04
Insular	30 (15.8%)	11 (25%)	19 (13.0%	NS
Corpus callosum	52 (27.4%)	18 (40.9%)	34 (23.3%)	.023
Extension				
Unilobar	92 (48.4%)	15 (34%)	77 (52.7%)	*ref*
Multilobar	98 (51.6%)	29 (66%)	69 (47.3%)	.03
Hemipshere^c^				
Right	90 (47.4%)	20 (45.5%)	70 (48%)	NS
Left	71 (37.4%)	12 (27.3%)	59 (40.4%)	.04
Midline cross				
No	161 (84.7%)	32 (72.7%)	129 (88.4%)	*ref*
Yes	29 (15.3%)	12 (27.3%)	17 (11.6%)	.01
Tumor characteristics				
Contrast enhancement	119 (62.6%)	34 (77.3%)	85 (58.2%)	.004
Mass effect	114 (60%)	31 (70.5%)	83 (59.9%)	NS
Edema	76 (40%)	24 (54.6%)	52 (35.6%)	.03
Intratumoral cyst	40 (21.1%)	11 (25%)	29 (19.9%)	NS
Calcification	57 (30%)	20 (45.5%)	37 (25.3%)	.01
Necrosis	32 (16.8%)	8 (18.2%)	24 (16.4%)	NS

Univariate analysis: logistic regression without covariate adjustment.

When patients presented seizures as the only clinical manifestation.

When unilateral; CS, classical survivors; F, female; KPS, Karnofsky performance status; M, male; NS, not significant; STS, short-term survivors; y, years.

**Figure 1.**
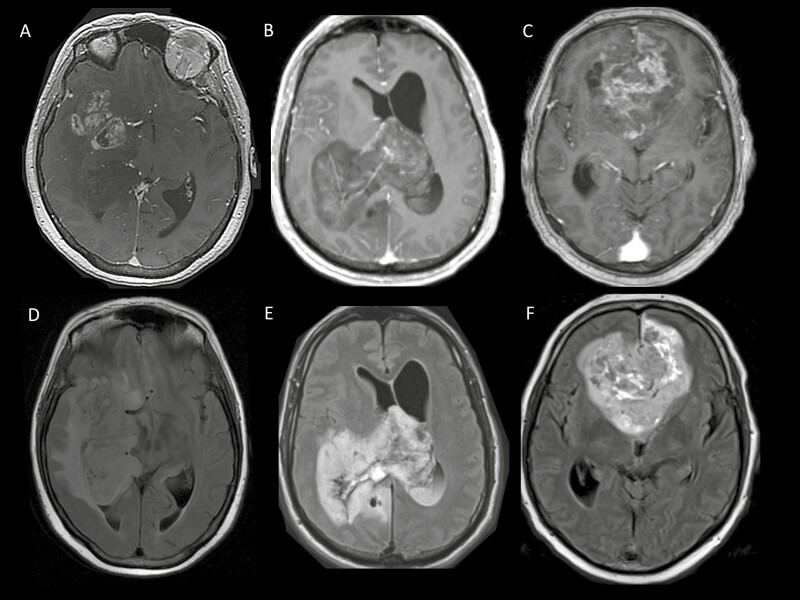
Representative examples of magnetic resonance imaging (MRI) presentation in 3 STS patients. Top: post-gadolinium axial T1-weighted images in 3 different STS patients (**A**, **B**, **C**) Bottom: corresponding axial Fluid-attenuated inversion recovery (FLAIR) (**D**, **E**, **F**).

In terms of radiological characteristics, compared to CS, tumors in STS more frequently presented with contrast enhancement (77.3% vs 58.2%; *P* = .= .004), edema (54.6% vs 35.6%; *P* = .= .03), and calcifications (45.5% vs 25.3%; *P* = .= .01). They were more frequently located in the parietal lobe and occipital lobe respectively (38.6% vs 17.8%; *P* = .= .005 and 18.2% vs 7.5%; *P* = .= .04) and they more frequently affected the corpus callosum (40.9% vs 23.3%, *P* = .= .023). They also more frequently involved multiple lobes (66% vs 47.3%; *P* = .= .03) and crossed the midline (27.3% vs 11.6%; *P* = .= .01) (Fig. 1). Consistently, the mean tumor volume was higher for STS (186 cm^3^) than for CS (90 cm^3^; *P* < .001) in the subset of patients for whom it could be assessed (STS *n* = 16 and CS *n* = 48).

### Histo-Molecular Characteristics

The histo-molecular of STS and CS is summarized in Table 2. Compared to CS, AO in STS were associated with a higher level of nuclear atypia (79.6% vs 48%; *P* = .= .0006), displayed a higher number of mitoses (10 vs 7; *P* = .= .02), a higher level of Ki67 expression (median 25% vs 15%; *P* = .= .0003), and a higher number of chromosome arm alterations (5.14 vs 3.76; *P* = .= .02). In addition, TP53 median expression was higher in STS (median 10.79% vs 3.92%; *P* = .= .01). Chromosome arm 9p loss and *CDKN2A* deletion were more frequent in STS than CS but the difference did not reach statistical significance.

**Table 2.** Comparison of STS and CS patients: histo-molecular characteristics.

	All patients, *n* (%)	STS,* n* (%)	CS,* n* (%)	*P*-values^a^
*N*	190	44	146	.0006
Morphology				
Nuclear atypia	105 (55.3%)	35 (79.6%)	70 (48%)	.02
Number of mitoses (median)	7	10	7	.004
Mitoses >8	61 (32.1%)	23 (57.5%)	38 (31.4%)	NS
Necrosis	41 (21.6%)	11 (25%)	30 (20.6%)	NS
MVP	147 (77.4%)	37 (84.1%)	110 (75.3%)	.0003
Immunohistochemistry				
KI67 expression (median)	20	25	15	.0001
Ki67<25 vs ≥25				.01
TP53 expression (median)	5.54	10.79	3.92	NS
TP53**-**positive expression	36 (18.9%)	10 (22.7%)	26 (17.8%)	NS
EGFR**-**positive expression	31 (16.3%)	5 (11.4%)	26 (17.8%)	NS
CIC loss	67 (35.3%)	18 (41%)	49 (33.6%)	NS
Genomic alterations				
Chr 9p loss	71 (37.4%)	19 (43.2%)	52 (35.6%)	NS
Chr 9q loss	30 (15.8%)	8 (18.2%)	22 (15.1%)	NS
Chr 7 gain	21 (11.1%)	5 (11.4%)	16 (11%)	NS
Chr 10q loss	22 (11.6%)	8 (18.2%)	14 (9.6%)	
*CDKN2A* deletion	14 (7.4%)	6 (13.6%)	8 (5.5%)	NS
Mean number of chr arm alteration (total)	4.08	5.14	3.76	.02
TERT promoter mutations^b^				
C228T	116 (72%)	30 (78%)	86 (69.9%)	NS
C250T	38 (23%)	6 (15.7%)	32 (26%)	NS
None	8 (4.9%)	2 (5.2%)	6 (4.8%)	NS

Univariate analysis: logistic regression without covariate adjustment.

TERT promoter mutation was not available in 29 tumors and 1 tumor presented both mutations.

Chr, chromosome; CS, classical survivors; MVP, microvascular proliferation; NS, not significant; STS, short-term survivors.

### Treatment

Treatment characteristics of STS and CS are summarized in Table 3. Short-term survivors less frequently underwent tumor resection than CS (61.9% vs 88.8%; *P* = .= .0001) and more frequently needed postoperative steroids (66% vs 42%; *P* = .= .009). Median time from surgery to postoperative treatment onset tended to be shorter in STS than in CS (47 vs 65 days, *P = .*08). After surgery, STS less frequently received the treatment that was considered as the standard treatment at the time of diagnosis than CS. Before 2012, they were less frequently treated with RT alone, and after 2012, less frequently treated with RT plus PCV (40.9% vs 63%, *P* = .= .01). Compared to CS, STS were more frequently treated with CT alone (22.7% vs 6.8%, *P* = .= .009) or radiotherapy plus TMZ (29.6% vs 15.7%, *P* = .= .05), and less frequently with RT alone (18.2% vs 49.3%, *P* = .= .0009).

**Table 3.** Comparison of STS and CS patients: treatments.

	All patients, *n* (%)	STS, *n* (%)	CS,* n* (%)	*P*- values^a^
*N*	190	44	146	.0001
Extent of resection^b^				
Biopsy	32 (16.8%)	16 (38.1%)	16 (11.2%)	
Surgery	153 (80.5%)	26 (61.9%)	127 (88.8%)	
Postoperative corticotherapy	91 (47.9%)	29 (65.9%)	62 (42.5%)	.009
Postoperative treatment				.0009
Radiotherapy alone	80 (42.1%)	8 (18.2%)	72 (49.3%)	
Chemotherapy alone	20 (10.5%)	10 (22.7%)	10 (6.8%)	
PCV	7 (3.7%)	5 (11.4%)	2 (1.4%)	
TMZ	13 (6.8%)	5 (11.4%)	8 (5.5%)	
Radiochemotherapy	75 (39.5%)	24 (54.6%)	51 (35%)	
PCV	36 (18.9%)	10 (22.7%)	26 (17.8%)	
TMZ	36 (18.9%)	13 (29.6%)	23 (15.7%)	
CCNU	1 (0.5%)	1 (2.3%)	0 (0.0%)	
TMZ plus BCNU	2 (1%)	0 (0%)	2 (1.4%)	
No treatment/**s**urveillance	15 (7.9%)	2 (4.5%)	13 (8.9%)	
Standard treatment	110 (57.9%)	18 (40.9%)	92 (63%)	.01
RT alone <2012		8/17 (47%)^c^	68/110 (62%)^c^	
RT+PCV≥2012		10/27 (37%)^d^	24/36 (66%)^d^	

Univariate analysis: logistic regression without covariate adjustment.

Extent of surgery was unavailable in 2 of the 44 STS and 3 of the 146 CS patients for technical reasons.

% calculated based on the number of STS (*n* = 17) and CS (*n* = 110) patients treated before 2012.

% calculated based on the number of STS (*n* = 27) and CS (*n* = 36) patients treated after 2012.

CS, classical survivors; STS, short-term survivors; NS, not significant; TMZ, temozolomide; PCV, lomustine + procarbazine + vincristine CCNU, lomustine; RT, radiotherapy.

### Multivariate Analysis

**Figure 2.**
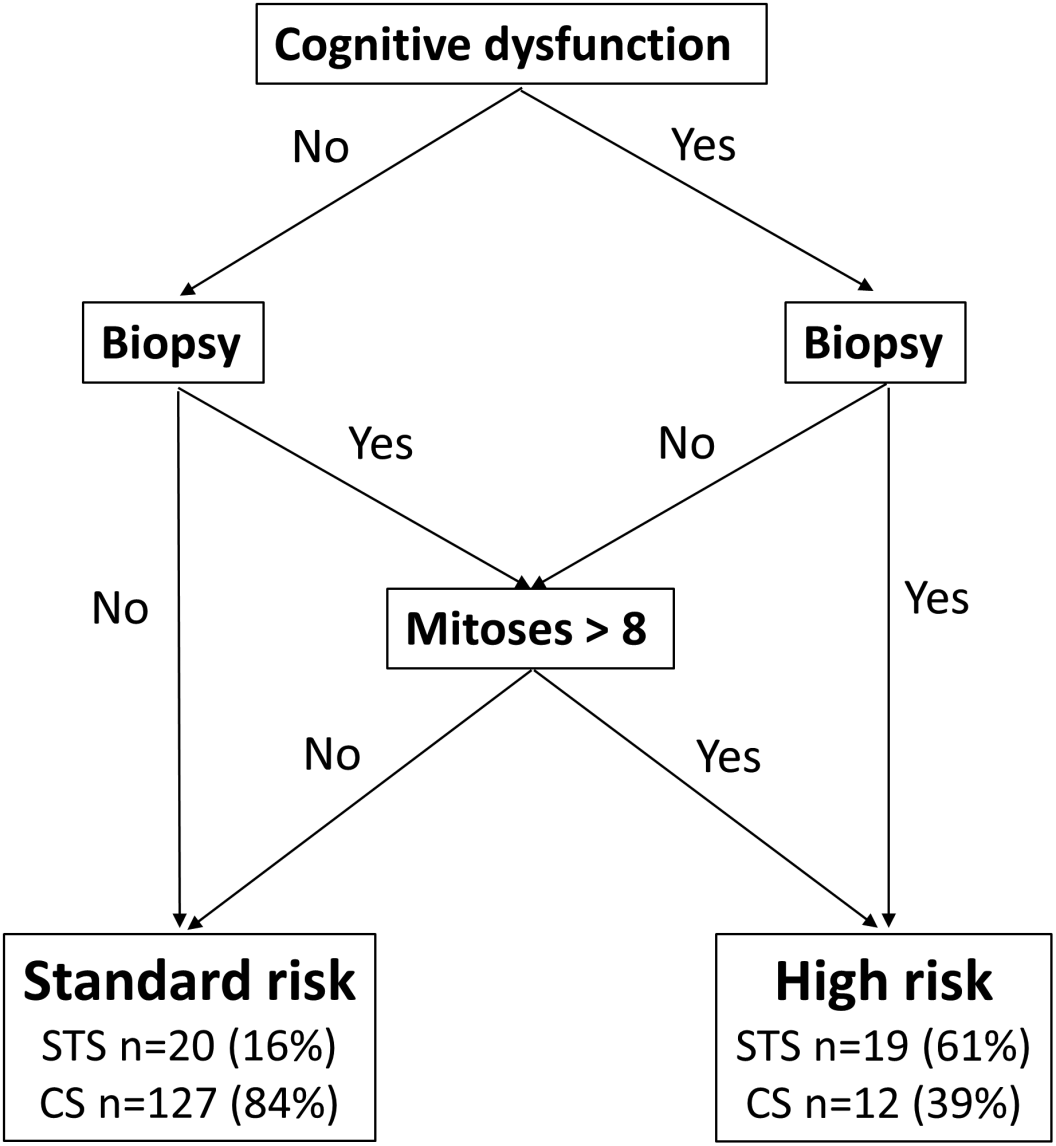
Diagram of patient classification based on the presence of cognitive dysfunction, extent of surgery, and number of mitoses.

The following characteristics were associated with the STS profile in multivariate analysis: the presence of cognitive dysfunction at diagnosis (odds ratio [OR] = 4.94; 95% confidence interval [CI] [2.02; 12.08]; *P* = .= .0005), the presence of a number of mitosis > 8 (OR = 0.25; 95% CI [0.10; 0.60]; *P* = .= .0022), and the absence of tumor resection (OR = 5.24; 95% CI [1.89; 14.51]; *P* = .= .0014; Table 4). Based on these 3 characteristics, patients could be classified into groups of standard (16%) and high (61%) risk of < 5 year survival (Fig. 2).

**Table 4.** Multivariate analyses of factors associated with STS.

	All patients, *n* (%)	STS, *n* (%)	CS,* n* (%)	OR [95% CI]	*P*-values^a^
*N*	190	44	146		
Cognitive dysfunction	52 (27.4%)	26 (59.1%)	26 (17.8%)	4.49 [2.02-12.08]	.0005
Extent of resection					
Surgery	153 (80.5%)	26 (61.9%)	127 (88.8%)	5.24 [1.89-14.51]	.0014
Biopsy	32 (16.8%)	16 (38.1%)	16 (11.2%)	*ref*	*ref*
Number of mitoses					
>8	61 (37.8%)	23 (57.5%)	38 (31.4%)	0.25 [0.10-0.60]	.0022
≤8	100 (62.2%)	17 (42.5%)	83 (68.6%)	*ref*	*ref*

Multivariate analysis: logistic regression without covariate adjustment.

CS, classical survivors; STS, short-term survivors; OR, odds ratio; CI, confidence interval.

## Discussion

Although AO are frequently associated with prolonged survival, approximately 20% of patients die within 5 years after diagnosis.^4-6^ The reason why some patients have a poor prognosis remains to be fully understood. Several studies have analyzed prognostic factors in AO but many of these studies included both 1p/19q codeleted and non-codeleted tumors as well as low-grade and high-grade oligodendrogliomas (Table 5). To our knowledge, our study is the first one to analyze the characteristics of STS. The present study showed that although the poor prognosis of STS appears to largely result from more aggressive baseline characteristics and a more advanced disease at diagnosis, surgical resection may be a particularly important determinant of survival in these patients.

**Table 5.** Summary of studies reporting prognostic factors in anaplastic oligodendrogliomas within the 10 last years.

Author, year	Study population	Number of patients	1p/19q codeletion (*n* = %)	Analysis restricted to 1p/19q codeleted AO	Characteristics associated with worse prognosis			^(Refs.)^
					Clinico-radiological	Histo-molecular	Treatment related	
Roux, 2020	O, AO	108	108 (100)	No	Growth rate ≥8 mm/year			16
Garton, 2020	O, AO	2514	1067 (42)	No	Older age, worse comorbidity index, infratentorial location	Grade III	No debulking, RT	17
Shin, 2020	AO	95	31 (32)	No		Ki67 (>20%), no *IDH* mutation		18
Lin, 2020	O, AO	186	186 (100)	No	Older age, lower KPS			19
Pouget, 2020	AO	220	220 (100)	Yes	Older age	MCM6 ≥ 50% or Ki67 ≥ 15%		20
Appay, 2019	AO, AA, GBM *IDH*m	911	483 (53)	Yes	Older age	CDKN2A deletion		21
Kinslow, 2019	O, AO	3135	unknown	No	Year of diagnosis, older age, sex (male), single status, infratentorial location	Grade III	No debulking, RT	22
Chen, 2019	O, AO	412	333 (80)	No	Older age, grade III	No 1p19q codeletion, 1p19q codeletion with polysomy	No GTR	23
Liu, 2019	AO	1899	Unknown	No	Older age, single status, multiple primary malignancies		No surgery	9
Yeboa, 2018	O, AO	1618	1618 (100)	No	Older age, worse comorbidity index, lower income	Astrocytoma/mixed glioma, grade III		24
Halani, 2018	O, AO	169	169 (100)	No	Older age	Grade III, PI3K mutations		25
Appay, 2018	AO	227	227 (100)	Yes	Older age	Necrosis, higher proliferative index, absence of SSTR2A expression		26
Rosenberg, 2018	AO	197	197 (100)	Yes	Older age	Necrosis, severe nuclear atypia, deletion of peak 9p21.3; amplification of peaks 14q13.1, 11q14.2, 20q13.33, and 1q21.3; CN‐LOH peak 17p11.2; gain of 11p		27
Aoki, 2018	O, AO, A, AA	414	164 (39)	No	Age (≥60)	Notch1 mutation	No GTR	28
Zetterling, 2017	O, AO	214	64 (30)	No	Older age, non-frontal location, neurological deficit or personality change	No IDH-mt and no 1p/19q codel		8
Figarella-Brnager, 2016	AO	157	157 (100)	Yes	Older age	MVP, necrosis, mitosis, KI67 LI		29
Hu, 2017	O, AO	374	374 (100)	No	Older age	Grade III, gene expression profile		30
Kamoun, 2016	AO, AA, GBM *IDH*m	156	80 (51)	No	Older age	Grade III, gene expression profile		31
Kang, 2015	AO	376	95 (25)	Yes	Age (>45), KPS (<70), non-frontal lobe location		No complete ­removal, no RT	32
Preusser, 2012	AO	281	76 (27)	No		Higher proliferative index		33
Lassman, 2011	AO	1013	301 (29)	No	Age (≥50), KPS (<70), non-frontal lobe location, bilateral hemispheric involvement	No 1p/19q codeletion	Biopsy, RT alone	7

AO, anaplastic oligodendroglioma; A, astrocytoma; AA, anaplastic astrocytoma; CN-LOH, copy-neutral loss of heterozygosity; GBM, glioblastoma; GTR, gross total resection; KPS, Karnofsky performance status; LI, label index; MVP, microvascular proliferation; O, oligodendroglioma; RT, radiotherapy.

### Baseline Characteristics of STS

Older age and poorer KPS have been identified as poor prognostic factors in AO across multiple studies and characterized STS in the present study.^7-9,17-32^ Being aged > 60 years was associated with STS, which is consistent with a large retrospective study reporting an approximately 2 times higher median survival in 1p/19q codeleted AO patients aged < 60 years compared to those aged > 60 years.^34^ In addition, we found that STS had a more aggressive clinical presentation than CS, with less frequent seizures, more frequent neurological and cognitive deficits, the latter feature was the only clinical feature independently associated with STS in multivariate analysis. Except in one study (which however included both low- and high-grade oligodendrogliomas and 1p/19q codeleted and non-codeleted tumors), the clinical presentation has not been related to prognosis in AO.^8^ In contrast, neurological deficits and the absence of seizures are well-described poor prognostic factors in low-grade gliomas.^35,36^ Most *IDH*-mutant glioma patients display seizures and it has been suggested that 2-hydroxyglutarate, the oncometabolite resulting from the *IDH* mutation, could explain their epileptogenicity.^37^ The reason why some *IDH*-mutant glioma patients do not display seizures remains to be determined but these patients could have a poorer outcome due to a longer time to diagnosis.

Regarding radiological characteristics, STS also had a more aggressive presentation than CS. Tumors in STS were larger and more frequently presented with contrast-enhancement. Consistently, contrast-enhancement has been associated with more aggressive molecular features in AO.^38^ Initial tumor volume has not been reported as a prognostic factor in AO; however, this finding is consistent with the reported poor prognostic value of bilateral hemispheric involvement, which was in the present study one of the radiological feature associated with STS.^7^

At the histo-molecular level, STS were characterized by a higher proliferative index and a number of mitosis > 8 was independently associated with STS in multivariate analysis. A higher proliferative index has been shown to be associated with a poorer outcome in AO^20,21,29,18,33^ and in a recent study a radiological growth rate > 8 mm/year has been found as an independent factor of poorer PFS in oligodendrogliomas.^35^ Higher proliferation index and reduced epileptogenicity could explain why STS presented larger and more symptomatic tumors in older patients. In addition, STS were characterized by a higher level of chromosomal instability compared to CS. In *IDH*-mutant astrocytomas, chromosomal instability is an established poor prognostic factor.^39^ Its prognostic value in AO remains to be fully established, yet it has been associated with more frequent contrast-enhancement, larger tumor volume, and a poorer prognosis.^23,38^ Chromosome 9p loss and *CDKN2A* deletion have been shown to be important poor prognostic factors in AO.^21^ Herein, there was a trend toward more frequent 9p loss in STS compared to CS, but this trend was not statistically significant, possibly because of the small sample size. Other molecular alterations that have been associated with poorer outcome in AO include *NOTCH1* and *PI3KCA* mutations, as well as specific gene expression profiles.^24,28^ Yet these alterations were not assessed in the present study. Future comprehensive molecular analyses will be important to determine whether STS are characterized by specific alterations that could facilitate their identification and constitute therapeutic targets.

### Treatment Characteristics of STS

Although we observed important differences regarding the treatment of STS and CS, the only treatment-related characteristic independently associated with STS in multivariate analysis was the absence of surgical resection. Surgical resection has been associated with better prognosis in several studies^7,9,17,22,23,28,32^ and the present study suggests that it may be particularly important in AO patients presenting aggressive baseline characteristics. The possibility of a surgical resection should therefore be reconsidered in AO patients who have only undergone a biopsy, possibly because a diagnosis of AO was not suspected pre-operatively. Indeed, approximately 20% of AO patients have a “glioblastoma-like” presentation that may lead some teams to perform a biopsy rather than a surgical resection, especially in older patients with cognitive dysfunction.^36^ After surgery, we observed that compared to CS, STS were more frequently treated with CT alone or with radiotherapy plus temozolomide. Older age and larger tumor volume could explain why STS were more frequently treated with CT alone than CS, while one can hypothesize that a more aggressive “glioblastoma-like” presentation could explain partly why STS were more frequently treated with radiotherapy plus temozolomide. Although the optimal treatment of patients at risk for poor outcome remains to be determined, CT alone, especially with temozolomide may not be the optimal treatment AO.^31,40^ Whether these patients benefit from the addition of CT to radiotherapy is also unclear. Indeed, in both the RTOG and EORTC trials, survival curves of patients treated with RT plus PCV or RT only started to diverge after 5 years, as if the addition of PCV to RT had no clear impact on the outcome of AO patients at risk for poor survival.^4,5^ Analysis of STS characteristics in ongoing clinical trials dedicated to AO (NCT00887146, NCT02444000) will be important to determine the impact of post-operative treatment in these patients.

### Identification of STS

Identification of patients at risk for poor survival is crucial to test more effective treatment strategies in this population. Herein, combining 3 characteristics independently associated with STS (cognitive dysfunction, mitosis count, and type of surgery) enabled to distinguish 2 groups of patients with different risk of short-term survival. However, this finding needs to be validated in an independent series and future studies should try to determine baseline features that are easier to assess for the identification of STS and explore classifications. Indeed, the identification of cognitive dysfunction may depend on testing method, mitosis count may lack reproducibility, and the type of surgery performed may depend on neurosurgeons’ experience.

### Study Limits

Our study is limited by the absence of volumetric analysis for all patients, the absence of in-depth molecular analyses, and the heterogeneity of post-operative treatments. Because of its retrospective design, it is also difficult to determine to what extent differences regarding treatment resulted from differences in baseline characteristics and to what extent they influenced the outcome. Despite these limits, our study provides the first description of STS characteristics, highlights features that could help identifying these patients, and suggests that surgical resection may be particularly important in this population. However, these findings require validation in independent series. In addition, although cancer-specific survival may be difficult to assess in retrospective studies, our study strongly suggests that future studies on this population should carefully analyze the cause of death in poor prognosis AO patients, since here approximately 20% of the patients who died < 5 years after diagnosis very likely died from an AO-unrelated cause. In a large series from the Surveillance, Epidemiology, and End Result (SEER) database, the rate of non-cancer death was 11.7% in adult oligodendrogliomas^20^ but this rate may be higher in the first years after AO diagnosis.

## Conclusions

The present study suggested that STS poor survival largely results from more aggressive baseline characteristics and a more advanced disease at diagnosis. In these patients, reduced epileptogenicity and a higher proliferation index could lead to the diagnosis of large and symptomatic tumors in older patients. Future studies will have to determine how to optimally identify and treat AO patients at risk for poor outcome, yet surgical resection may be particularly important in this population.

## Supplementary Material

oyac023_suppl_Supplementary_Figure

## Data Availability

The data underlying this article will be shared on reasonable request to the corresponding author.
