# Supplementary figures and images for: Characteristics of Anaplastic Oligodendrogliomas Short-Term Survivors: A POLA Network Study
Source: Oncologist. 2022 Mar 5;27(5):414–23. doi: 10.1093/oncolo/oyac023 (PMC9074983; doi:10.1093/oncolo/oyac023)

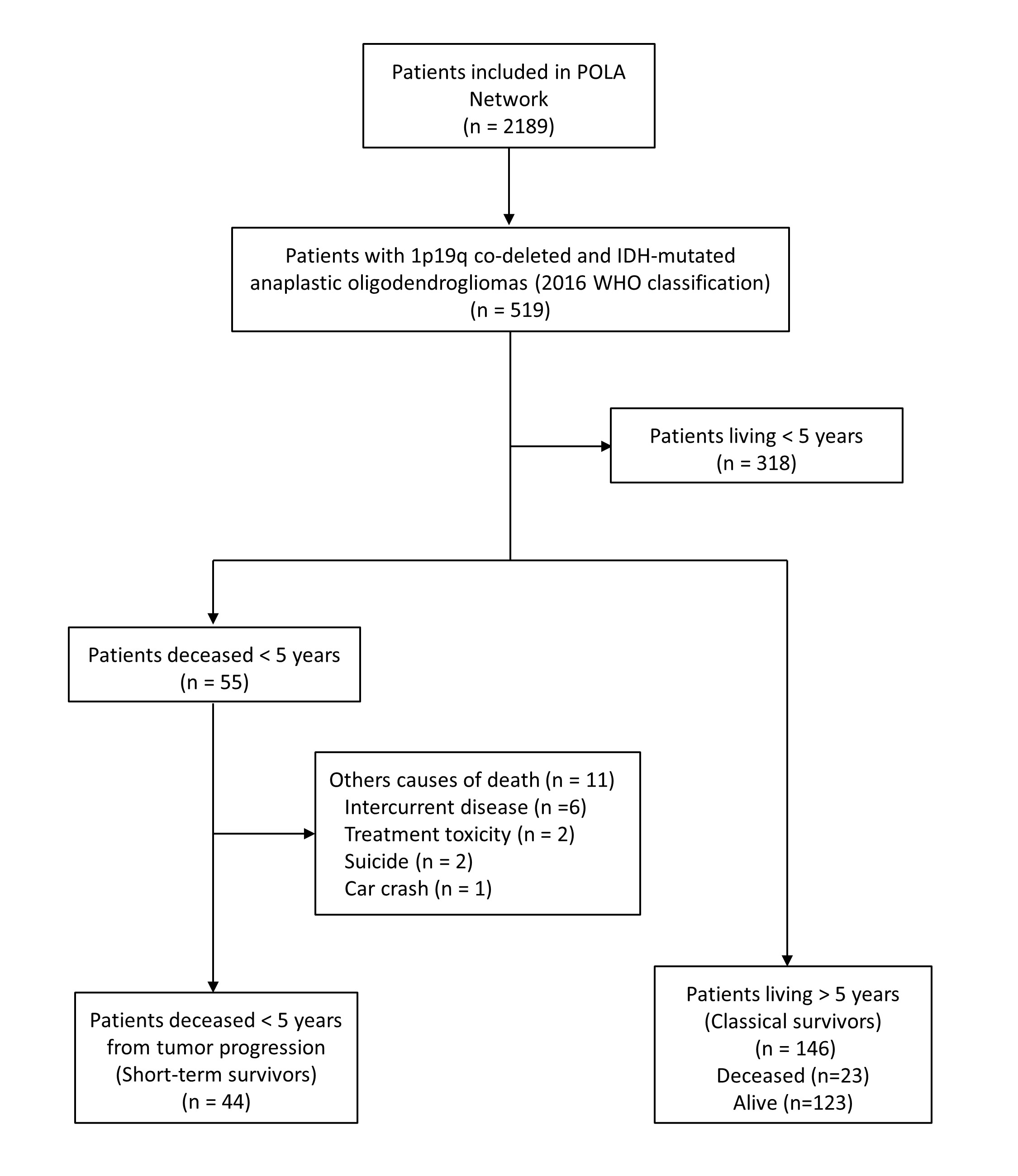

Supplement: oyac023_suppl_Supplementary_Figure [file oyac023_suppl_supplementary_figure.jpeg]
